# Sex differences in a mouse model of multiple sclerosis: neuropathic pain behavior in females but not males and protection from neurological deficits during proestrus

**DOI:** 10.1186/2042-6410-5-4

**Published:** 2014-02-28

**Authors:** Elizabeth J Rahn, Tommaso Iannitti, Renee R Donahue, Bradley K Taylor

**Affiliations:** 1 Department of Physiology, University of Kentucky, 800 Rose Street, Lexington, KY 40536, USA; 2 Present Address: Department of Neurobiology, University of Alabama at Birmingham, 1825 University Blvd. SHEL 1070C, Birmingham, AL 35294, USA; 3 Present Address: School of Biomedical Sciences, University of Leeds, Leeds LS2 9JT, UK

**Keywords:** Experimental autoimmune encephalomyelitis, Estrous, MOG_35-55_, Proestrus

## Abstract

**Background:**

Multiple sclerosis (MS), a demyelinating disease of the central nervous system, is one of the most prevalent neurological disorders in the industrialized world. This disease afflicts more than two million people worldwide, over two thirds of which are women. MS is typically diagnosed between the ages of 20–40 and can produce debilitating neurological impairments including muscle spasticity, muscle paralysis, and chronic pain. Despite the large sex disparity in MS prevalence, clinical and basic research investigations of how sex and estrous cycle impact development, duration, and severity of neurological impairments and pain symptoms are limited. To help address these questions, we evaluated behavioral signs of sensory and motor functions in one of the most widely characterized animal models of MS, the experimental autoimmune encephalomyelitis (EAE) model.

**Methods:**

C57BL/6 male and female mice received flank injection of complete Freund’s adjuvant (CFA) or CFA plus myelin oligodendrocyte glycoprotein 35-55 (MOG_35-55_) to induce EAE. Experiment 1 evaluated sex differences of EAE-induced neurological motor deficits and neuropathic pain-like behavior over 3 weeks, while experiment 2 evaluated the effect of estrous phase in female mice on the same behavioral measures for 3 months. EAE-induced neurological motor deficits including gait analysis and forelimb grip strength were assessed. Neuropathic pain-like behaviors evaluated included sensitivity to mechanical, cold, and heat stimulations. Estrous cycle was determined daily via vaginal lavage.

**Results:**

MOG_35-55_-induced EAE produced neurological impairments (i.e., motor dysfunction) including mild paralysis and decreases in grip strength in both females and males. MOG_35-55_ produced behavioral signs of neuropathic pain—mechanical and cold hypersensitivity—in females, but not males. MOG_35-55_ did not change cutaneous heat sensitivity in either sex. Administration of CFA or CFA + MOG_35-55_ prolonged the time spent in diestrus for 2 weeks, after which normal cycling returned. MOG_35-55_ produced fewer neurological motor deficits when mice were in proestrus relative to non-proestrus phases.

**Conclusions:**

We conclude that female mice are superior to males for the study of neuropathic pain-like behaviors associated with MOG_35-55_-induced EAE. Further, proestrus may be protective against EAE-induced neurological deficits, thus necessitating further investigation into the impact that estrous cycle exerts on MS symptoms.

## Background

Multiple sclerosis (MS) is an autoimmune demyelinating disease of the central nervous system (CNS) that afflicts twice the number of women as compared to men [1]. Sex has been shown to impact both the pathology and severity of MS. While the immune response associated with MS is more robust in women, the pathology and prognosis for men are generally associated with more progressive neurodegeneration [2]. Despite this large sex disparity in disease prevalence and severity, the impact of estrous cycle and correlated alterations in circulating ovarian hormones (estrogens/progestogens) on MS has yet to be fully characterized. Limited reports indicate that clinical manifestations of MS (e.g., weakness, numbness, tingling, and tiredness) tend to increase when ovarian hormones are low and decrease when they are high [3-5]. Additionally, pregnancy-associated remission of symptoms is believed to be associated with increased circulating ovarian hormones [6,7].

Although neurological deficits such as muscle weakness, spasticity, and paralysis are considered canonical symptoms of MS, chronic pain is a symptom frequently experienced by patients which can antedate other neurological impairments. Pain is considered a primary factor in the suffering and poor quality of life experienced by MS patients, with an overall prevalence of approximately 63% [8-11]. Cardinal features of neuropathic pain in multiple sclerosis include hypersensitivity to cutaneous mechanical and thermal stimuli in the distal extremities [10,12-14]. Sex differences in pain symptoms of MS is an understudied area of research, and the literature yields limited and conflicting results. Some clinical studies suggest that pain is worse in females [15,16], while others report no differences [17-19].

The serendipitous advent of animal models mimicking the pathology and symptoms of MS has allowed basic researchers to investigate disease progression, symptomology, and therapeutic interventions. These widely utilized models include the experimental autoimmune encephalomyelitis (EAE) and Theiler’s murine encephalomyelitis virus (TMEV) models. Research evaluating sex differences in both models have yielded variable results, undoubtedly influenced by a number of factors including autosomal genotype of murine strains and the immunization agent/protocol used [20-23]. Although a number of studies characterize manipulation of ovarian hormones in these models, we were unable to find a single study evaluating natural estrous cycle and corresponding alterations in motor impairments, disease progression, or any other behavioral measure. Preclinical animal models of MS virtually ignore the question of neuropathic pain-like behaviors in males versus females with the exception of two reports indicating that allodynia and/or hyperalgesia is more pronounced in females [22,24].

The present study was designed to investigate the effects of sex and estrous state on the development, duration, and severity of neurological deficits and neuropathic pain-like behavior in a mouse EAE model induced by myelin oligodendrocyte glycoprotein 35–55 (MOG_35-55_) [25]. The MOG_35-55_ EAE model has previously been shown to produce robust and reproducible motor dysfunction, demyelination, and neuropathic pain-like behaviors [26-28]. We hypothesized that while both males and females would demonstrate development of neurological impairments (i.e., motor dysfunction) typical of EAE, females would show more severe neuropathic pain-like behaviors. Based on the clinical findings of reduced MS symptoms during the luteal phase and remission of MS symptoms during pregnancy, we further hypothesized that these symptoms would be attenuated during the proestrus phase, when circulating ovarian hormones peak.

## Methods

### Animals

This study used 80 C57BL/6 mice purchased from Charles Rivers (Indianapolis, IN, USA), aged 12–14 weeks when the studies began. Mice were housed four to a cage, maintained in a temperature- and humidity-controlled environment on a 14/10 h light/dark cycle (lights on 4:00 a.m., lights off 6:00 p.m.). Since the estrous phase is stimulated by the presence of male pheromones [29,30], male and female cages were interspersed with each other. We did not control for pheromone exposure as the primary purpose was to keep female mice cycling; however, care was taken to spatially intersperse cages of females with equal numbers of cages housing male mice. Food and water were available *ad libitum*. Animals were allowed a minimum of 1 week to habituate to the facility prior to their entrance into the study. All animal procedures were approved by the Institutional Animal Care and Use Committee of the University of Kentucky, followed the guidelines for the treatment of animals of the International Association for the Study of Pain, and conducted in full compliance with the Association for Assessment and Accreditation of Laboratory Animal Care (AAALAC).

### General experimental methods

Two experimental studies were completed. Experiment 1 was designed to characterize sex differences observed in the EAE model induced by immunization with MOG_35-55_ (detailed protocol below). In this study, female and male C57BL/6 mice were immunized with complete Freund’s adjuvant (CFA) (females, *n* = 13; males, *n* = 10) or CFA + MOG_35-55_ (females, *n* = 11; males, *n* = 11) and evaluated behaviorally for neurological motor deficits and alterations to tactile and thermal stimulations for 21 days post-immunization. Experiment 2 evaluated the impact of estrous cycle on neurological motor impairments and responses to tactile and thermal stimulation in the EAE model. In experiment 2, female C57BL/6 mice were immunized with either CFA (*n* = 17) or CFA + MOG_35-55_ (*n* = 18) and evaluated for 43 days post-immunization. Estrous cycle was determined daily via vaginal lavage from day -7 through day 43 (detailed protocol below). To evaluate the duration of neuropathic pain-like behavior in the EAE model, a randomly chosen subset of animals from experiment 2 (CFA, *n* = 8; CFA + MOG_35-55_, *n* = 9) were tested for an additional period, from days 60–90 post-immunization.

All animals were weighed and evaluated daily for alterations in neurological motor function using a clinical assessment scoring system as previously described [31] (experiment 1: day -2 through day 21; experiment 2: day -7 through day 43, days 60, 75, and 90). Baseline grip strength, as well as mechanical and cold stimulations, was evaluated on day -2 for both experiments 1 and 2. Baseline thermal responses were measured on day -1 for both experiments. Immunization with either CFA or CFA + MOG_35-55_ occurred on days 0 and 6. Alterations in grip strength and responsivity to mechanical and cold stimulation were evaluated on days -2, 1, 3, 5, 7, 9, 11, 13, 17, and 21 in both experiments and continued on days 25, 29, 35, 42, 60, 75, and 90 for animals in experiment 2. Heat sensitivity was assessed on days -1, 4, 10, and 16 in both experiments and continued with days 28, 34, and 43 in experiment 2. Following the induction of EAE, which produces mild to moderate motor dysfunction, mice were given access to DietGel® 76A (Clear H_2_O®, Portland, OR, USA) in the bottom of cages to ensure that they maintained body weight. In experiment 1, males and females were tested concurrently but on different behavioral testing platforms that had been thoroughly cleaned with MB-10 (Quip Laboratories, Inc., Wilmington, DE, USA).

### Induction of EAE

EAE was induced with an immunization protocol utilizing MOG_33-55_ (AnaSpec Inc., Fremont, CA, USA), which leads to T cell infiltration in the central nervous system, in combination with pertussis toxin (List Biological Laboratories, Campbell, CA, USA), an agent which enhances EAE severity and disease onset (for review, see [32]). MOG_33-55_ was emulsified in a 1:1 solution of 1× phosphate-buffered saline (Fisher Scientific, Pittsburgh, PA, USA) and CFA. CFA was prepared at a concentration of 5 mg/ml of mycobacterium tuberculosis (Voigt Global Distribution, Lawrence, KS, USA) in incomplete Freund’s adjuvant (IFA, Sigma-Aldrich, St. Louis, MO, USA). On the afternoon of day 0 following behavioral assessment, MOG_35-55_ (150 μg, s.c. per flank) was bilaterally injected (100 μl) at the flank of each hindlimb under light isoflurane (1.5%–3% in oxygen; Butler Schein, Dublin, OH, USA) anesthesia (experiment 1) or gentle manual restraint (experiment 2). A booster injection of MOG_35-55_ (150 μg, s.c. per flank) was administered on day 6. Pertussis toxin was injected (200 ng/200 μl, i.p.) on days 0 and 2. Age- and sex-matched controls received identical treatment but were immunized with CFA only. Emulsification of MOG_35-55_ in CFA is necessary for the immunological response observed in canonical EAE models [33]. Therefore, although CFA is known to produce an inflammatory and pain response, this control group was critical as it allowed for differential pain behavior to be attributed to MOG_35-55_ rather than CFA immunization, thereby increasing our understanding of behaviors associated with the pathophysiology of experimental autoimmune encephalomyelitis.

### Behavioral assessment of sensory and motor functions

Fluctuations in room noise, vibrations, and temperature were minimized so as to facilitate acclimation and response reliability. Prior to sensory testing, the mice were acclimated for 30 min/day for 3 days to individual Plexiglas (10.16 × 10.16 × 25.4 cm) chambers. These boxes were placed on either an elevated stainless steel wire mesh (for tests of cold and mechanical sensitivity) or a Plexiglas floor (for tests of heat sensitivity). An additional habituation period of at least 30 min was provided before data collection on each testing day. Mechanical testing was performed prior to cold testing, and a minimum of 1 h was allowed between tests. Heat testing and cold/mechanical testing were conducted on alternating days to avoid sensitization. All behavioral measurements and injections were performed by a single experimenter (EJR). Animals were assigned numbers which did not indicate group condition. Coded testing sheets were used throughout behavioral testing to keep the experimenter blind to condition.

#### Mechanical sensitivity

Mechanical sensitivity was assessed using a digital electronic von Frey Anesthesiometer (model Alemo 2450; IITC Life Science, Woodland Hills, CA, USA), connected to a 90-g probe equipped with a flexible tip. The tip was applied to the plantar surface of the paw until paw withdrawal. Duplicate determinations were measured for each paw and averaged. A minimum inter-trial interval of 2 min was allowed to elapse between evaluations of paws. The testing took place in the following order: right, right, left, and left.

#### Cold sensitivity

Cold allodynia was assessed following the application of an acetone drop to the plantar surface of the hind paw as previously described [34]. Acetone was loaded into a syringe barrel, and air bubbles were cleared from the syringe prior to acetone application. One drop of acetone (approximately 10–12 μl) was applied through the mesh platform onto the plantar surface of the hind paw. Care was taken to gently apply the bubble of acetone (and not the tip of the applicator) to the plantar skin. The duration of time the animal shook, licked, or completely lifted its paw off the floor was recorded. The duration of paw withdrawal was recorded with a 60-s cutoff. Three observations were taken for each paw and averaged. A minimum inter-trial interval of 5 min was allowed to pass between observations for each pair of paws (i.e., right and left). The testing took place in the following order: right, left, right, left, right, and left.

#### Heat sensitivity

Mice were tested for sensitivity to heat using a radiant heat paw-withdrawal (Hargreaves) device [35]. The thermal stimulus consisted of a radiant heat source (8 V, 50-W lamp, Ugo Basile, Comerio, Italy) positioned under the glass floor directly beneath the hind paw. When triggered, a timer was activated, and light passed through a small aperture at the top of a movable case. One day before testing, voltage intensity was adjusted to standardize the average paw withdrawal latency at 10 ± 2 s. At specified time points, paw withdrawal latencies were measured in duplicate for each paw. A minimum inter-trial interval of 5 min was allowed between evaluations of the paws. Testing took place with the following order: right, right, left, and left. If the mouse did not respond within 30 s, the heat was discontinued to prevent tissue damage.

#### Neurological motor function

We monitored animals as they walked across a flat plane and checked their righting reflex after turning them over. Responses were scored according to the following clinical assessment scale [31]: grade 0, absence of clinical signs; grade 1, hanging tail or impaired righting; grade 2, mild paresis of one hind limb; grade 3, paresis of two hind limbs; and grade 4, full paralysis of one or two hind limbs/moribund.

#### Neuromuscular function

Neuromuscular function of the forelimbs was tested with a grip strength meter (Columbus Instruments, Columbus, OH, USA). The meter was positioned horizontally, and the mice were held by the tail and lowered toward the apparatus. The mice were allowed to grasp the smooth metal grid (forelimbs only) and were then pulled backward in the horizontal plane. The force applied to the grid at the moment the grasp was released was recorded as the peak tension (Newtons). Grip strength was measured in triplicate and averaged.

### Estrous cycle and vaginal lavage

Estrous cycle was monitored daily for female mice in experiment 2, from 7 days before through 43 days after MOG_33-55_ and/or CFA. Vaginal lavage was performed between 6 and 8 a.m. using gentle manual restraint. Animals were returned to their home cages following lavage and allowed approximately 2 h prior to behavioral evaluations, sufficient time to allow any stress-induced analgesia to subside. To collect vaginal cells, a glass Pasteur pipette (14.6 cm, Fischer Scientific, Pittsburg, PA, USA) that had been pulled over a flame to create an angled tip with a narrow opening was used. The tip of the pipette was fire-polished and examined under a microscope to confirm it was free of jagged edges. The pipette was attached to a bulb and filled with 100–200 ul of 0.9% saline (Sigma-Aldrich). The pipette tip was gently pressed against the vaginal opening, and the saline was slowly forced into the vagina (approximately 5–7 s) and withdrawn over several repetitions to obtain a representative sample of vaginal cells. Samples were placed in a 96-well plate, and classification was made using a Nikon Diaphot 300 microscope (Melville, NY, USA). On rare occasions, lavage did not yield enough cells for classification, and this is reflected in varying degrees of freedom in the statistics for experiment 2 comparing behavioral measures across estrous phases. The cycle stage was classified as estrus (cornified), proestrus (nucleated cells), diestrus (leukocyte cells), or metestrus (mixture of cells from various stages) as previously described [36,37]. Early time points in the study (prior to day 14) were associated with prolonged diestrus which resulted in decreased numbers of animals within estrus, proestrus, and metestrus as would have been present otherwise with normal cycling.

### Statistical analyses

Data were analyzed using analysis of variance (ANOVA) for repeated measures, two-way or one-way ANOVA as appropriate.

#### Experiment 1

**Figure 1:**
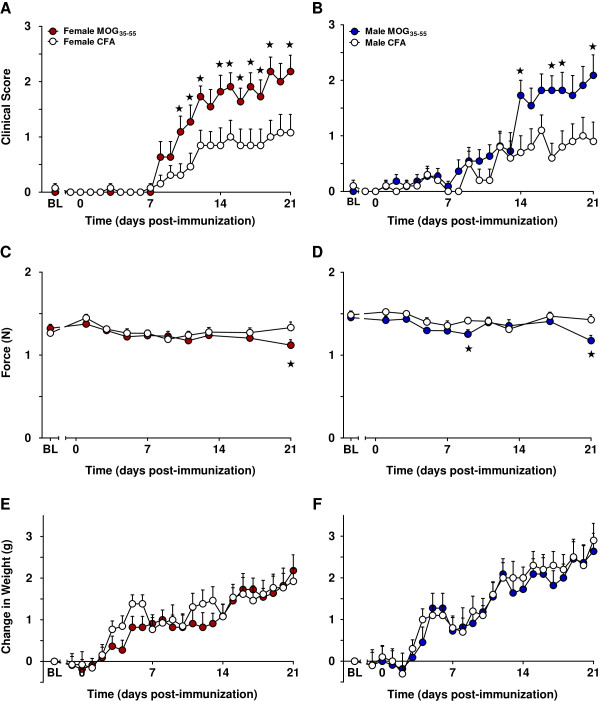
**EAE-induced neurological motor deficits are similar between 3–4-month-old female and male C57BL/6 mice.** Administration of the EAE-inducing agent myelin oligodendrocyte glycoprotein 35–55 (MOG_35-55_) was associated with neurological motor deficits as assessed with a clinical scoring system in female **(A)** and male **(B)** mice. Weak effects were also observed on grip strength (**C** and **D**, but see Figure 3B for significant effects at later time points). No differences in body weight were observed between the animals that received MOG_35-55_ versus CFA in either female **(E) **or male** (F)** mice. MOG_35-55_ injections began on day 0. *BL* baseline. Values represent mean ± SEM. ★*P* < 0.05 compared to sex- and age-matched CFA control (one-way ANOVA). *N* = 10–13/group.

**Figure 2:**
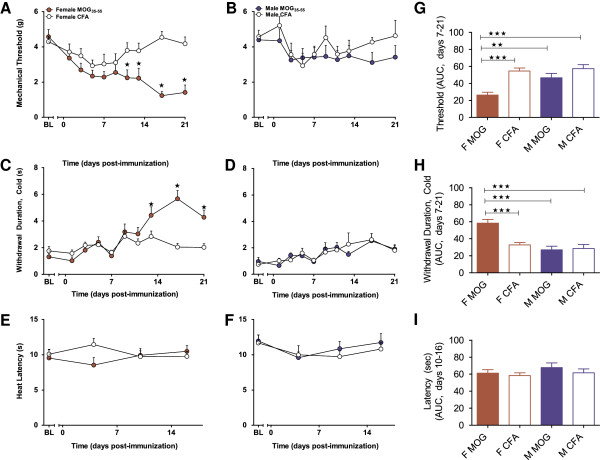
**EAE induces mechanical and cold hypersensitivity in female, but not male C57BL/6 mice.** Administration of MOG_35-55_ increased sensitivity to mechanical and cold stimulation in females (**A** and **C**, respectively), but not their male counterparts (**B** and **D**, respectively) relative to CFA sex- and age-matched controls. MOG_35-55_ did not alter paw withdrawal latencies in response to radiant heat in either female **(E)** or male **(F)** mice as compared to CFA age- and sex-matched controls. Area under the curve (AUC) analyses on days 7–21 examining sensitivity to mechanical **(G)** and cold **(H)** and days 10–16 examining thermal stimulation **(I)** in females *(F)* and males (*M*). MOG_35-55_ injections began on day 0. *BL* baseline. Values represent mean ± SEM. ★★★*P* < 0.001, ★★*P* < 0.01, and ★*P* < 0.05 compared to sex- and age-matched CFA control (one-way ANOVA) or comparison as indicated in AUC figures (one-way ANOVA and Bonferroni Correction). *N* = 10–13/group.

Three-way repeated measures (RM) ANOVAs were performed to determine the effect of sex (females vs. males) and treatment (MOG_35-55_ vs. CFA) over time. Following these analyses, two-way RM ANOVAs were performed to examine the effects of treatment (MOG_35-55_ vs. CFA) and time (Table 1). In cases where a main effect of treatment or interaction of treatment by time was observed with the two-way RM ANOVA, one-way ANOVAs were subsequently performed, comparing the treatment conditions at each time point. Area under the curve (AUC) transformations were performed on days 7–21 (mechanical/cold) and days 10–16 (heat) to correspond with the first behavioral assessment following the final MOG_35-55_ immunization through the final testing day. AUC transformations were performed to differentiate the effects of MOG_35-55_ from those of the CFA control condition and to graphically illustrate changes observed in female MOG_35-55_ mice relative to same-sex controls and male mice from both treatment conditions (Figures 1 and 2).

**Table 1:** Statistics for experiment 1

**Measure**	**Two-way RM ANOVA (treatment × time)**				**Three-way RM ANOVA (treatment × sex × time)**
	**Females**		**Males**		
	**Figure**		**Figure**		
Clinical scores	Figure 1A	Day: *F*_23,506_ = 25.1, *P* < 0.001	Figure 1B	Day: *F*_23,437_ = 14.3, *P* < 0.001	Day: *F*_23,943_ = 37.1, *P* < 0.001
		Treatment: *F*_1,22_ = 9.0, *P* < 0.01		Treatment: *F*_1,19_ = 8.6, *P* < 0.01	Treatment: *F*_1,41_ = 16.9, *P* < 0.001
		Day × treatment: *F*_23,506_ = 3.2, *P* < 0.05		Day × treatment: *F*_23,437_ = 2.2, *P* < 0.01	Sex: *F*_1,41_ = 0.5, *P* = 0.4
					Treatment × sex: *F*_1,41_ = 0.2, *P* = 0.6
					Day × treatment: *F*_23,943_ = 2.8, *P* < 0.01
					Day × sex: *F*_23,943_ = 1.3, *P* = 0.2
					Day × sex × treatment: *F*_23,943_ = 0.4, *P* = 0.8
Grip strength	Figure 1C	Day: *F*_9,198_ = 3.3, *P* < 0.001	Figure 1D	Day: *F*_9,171_ = 3.7, *P* < 0.001	Day: *F*_9,369_ = 5.8, *P* < 0.001
		Treatment: *F*_1,22_ = 2.0, *P* = 0.17		Treatment: *F*_1,19_ = 7.3, *P* < 0.05	Treatment: *F*_1,41_ = 8.2, *P* < 0.01
		Day × treatment: *F*_9,198_ = 1.2, *P* = 0.3		Day × treatment: *F*_9,171_ = 1.3, *P* = 0.2	Sex: *F*_1,41_ = 32.7, *P* < 0.001
					Treatment × sex: *F*_1,41_ = 0.7, *P* = 0.4
					Day × treatment: *F*_9,369_ = 1.9, *P* < 0.05
					Day × sex: *F*_9,369_ = 1.2, *P* = 0.2
					Day × sex × treatment: *F*_9,369_ = 0.6, *P* = 0.7
Weight	Figure 1E	Day: *F*_22,484_ = 16.0, *P* < 0.001	Figure 1 F	Day: *F*_22,418_ = 49.7, *P* < 0.001	Day: *F*_22,902_ = 54.4, *P* < 0.001
		Treatment: *F*_1,22_ = 0.2, *P* = 0.6		Treatment: *F*_1,19_ = 0.06, *P* = 0.8	Treatment: *F*_1,41_ = 0.2, *P* = 0.6
		Day × treatment: *F*_22,484_ = 0.8, *P* = 0.5		Day × treatment: *F*_22,418_ = 0.6, *P* = 0.9	Sex: *F*_1,41_ = 1.4, *P* = 0.2
					Treatment × sex: *F*_1,41_ = 0.005, *P* = 0.9
					Day × treatment: *F*_22,902_ = 0.6, *P* = 0.7
					Day × sex: *F*_22,902_ = 3.2, *P* < 0.01
					Day × sex × treatment: *F*_22,902_ = 0.7, *P* = 0.6
Mechanical thresholds	Figure 2A	Day: *F*_9,198_ = 4.2, *P* < 0.001	Figure 2B	Day: *F*_9,171_ = 2.0, *P* < 0.05	Day: *F*_9,369_ = 4.8, *P* < 0.001
		Treatment: *F*_1,22_ = 13.8, *P* < 0.01		Treatment: *F*_1,19_ = 1.3, *P* = 0.2	Treatment: *F*_1,41_ = 10.2, *P* < 0.01
		Day × treatment: *F*_9,198_ = 4.5, *P* < 0.001		Day × treatment: F_9,171_ = 0.5, *P* = 0.8	Sex: *F*_1,41_ = 7.3, *P* < 0.05
					Treatment × sex: *F*_1,41_ = 1.6, *P* = 0.2
					Day × treatment: *F*_9,369_ = 2.8, *P* < 0.01
					Day × sex: *F*_9,369_ = 0.8, *P* = 0.5
					Day × sex × treatment: *F*_9,369_ = 1.1, *P* = 0.3
Acetone withdrawal	Figure 2C	Day: *F*_9,198_ = 14.5, *P* < 0.001	Figure 2D	Day: *F*_9,171_ = 7.0, *P* < 0.001	Day: *F*_9,369_ = 20.2, *P* < 0.001
		Treatment: *F*_1,22_ = 6.5, *P* < 0.05		Treatment: *F*_1,19_ = 0.02, *P* = 0.8	Treatment: *F*_1,41_ = 2.7, *P* = 0.16
		Day × treatment: *F*_9,198_ = 8.2, *P* < 0.001		Day × treatment: *F*_9,171_ = 0.6, *P* = 0.6	Sex: *F*_1,41_ = 22.5, *P* < 0.001
					Treatment × sex: *F*_1,41_ = 3.4, *P* = 0.07
					Day × treatment: *F*_9,369_ = 4.3, *P* < 0.001
					Day × sex: *F*_9,369_ = 1.6, *P* = 0.13
					Day × sex × treatment: *F*_9,369_ = 5.2, *P* < 0.001
Heat latency	Figure 2E	Day: *F*_3,66_ = 0.06, *P* = 0.9	Figure 2F	Day: *F*_3,57_ = 1.8, *P* = 0.14	Day: *F*_3,123_ = 1.052, *P* = 0.3
		Treatment: *F*_1,22_ = 0.7, *P* = 0.3		Treatment: *F*_1,19_ = 0.2, *P* = 0.5	Treatment: *F*_1,41_ = 0.01, *P* = 0.8
		Day × treatment: *F*_3,66_ = 1.8, *P* = 0.15		Day × treatment: *F*_3,57_ = 0.2, *P* = 0.8	Sex: *F*_1,41_ = 2.1, *P* = 0.14
					Treatment × sex: *F*_1,41_ = 0.9, *P* = 0.3
					Day × treatment: *F*_3,123_ = 1.5, *P* = 0.19
					Day × sex: *F*_3,123_ = 1.1, *P* = 0.3
					Day × sex × treatment: *F*_3,123_ = 0.3, *P* = 0.8
AUC	Figure 2G	Mechanical thresholds: one-way ANOVA: *F*_3,41_ = 11.1, *P* < 0.001	Figure 2H	Acetone withdrawal: one-way ANOVA: *F*_3,41_ = 13.2, *P* < 0.001	Heat latency: one-way ANOVA: *F*_3,41_ = 0.8, *P* = 0.4 (Figure 2I)

Treatment conditions: CFA (females *n* = 13, males *n* = 10), CFA + MOG_35-55_ (females *n* = 11, males *n* = 11). *AUC* area under the curve.

#### Experiment 2

**Figure 3:**
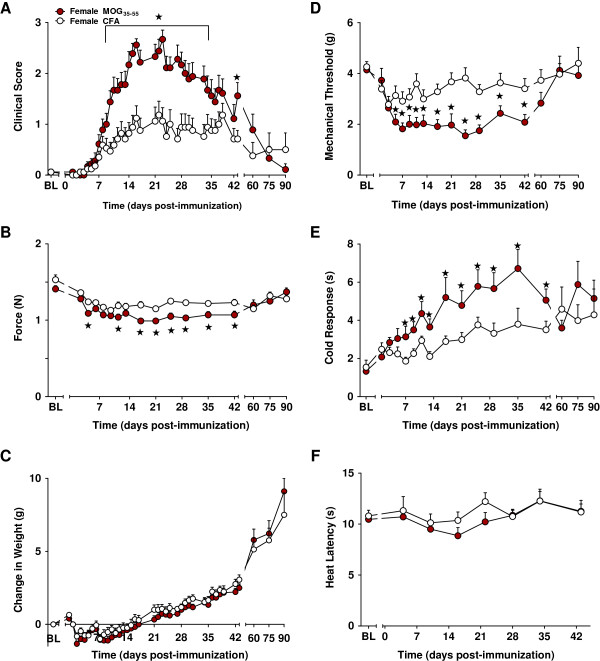
**EAE-induced neuropathic pain-like behaviors persist for 6 weeks in female C57BL/6 mice.** Administration of MOG_35-55_ increased neurological motor deficits **(A)** and decreased forelimb grip strength **(B)**, but did not change weight gain **(C)** relative to CFA controls. Female mice that received MOG_35-55_ displayed decreased mechanical withdrawal thresholds **(D)** and increased duration of response to acetone stimulation **(E)** when compared to CFA-treated controls. Response to heat stimulation did not differ between mice that received MOG_35-55_ or CFA alone **(F)**. MOG_35-55_ injections began on day 0. *BL* baseline. Values represent mean ± SEM. ★*P* < 0.05 compared to sex- and age-matched CFA control (one-way ANOVA). *N* = 8–18/group.

**Figure 4:**
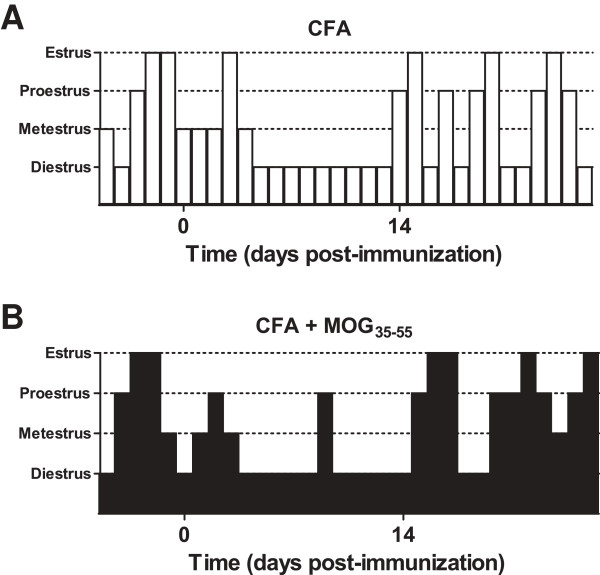
**CFA transiently increased the time spent in diestrus.** Representative traces of female mice that received either CFA **(A)** or CFA + MOG_35-55_**(B)**. Note that estrous cycling resumed 2 weeks after CFA or CFA + MOG_35-55_.

**Figure 5:**
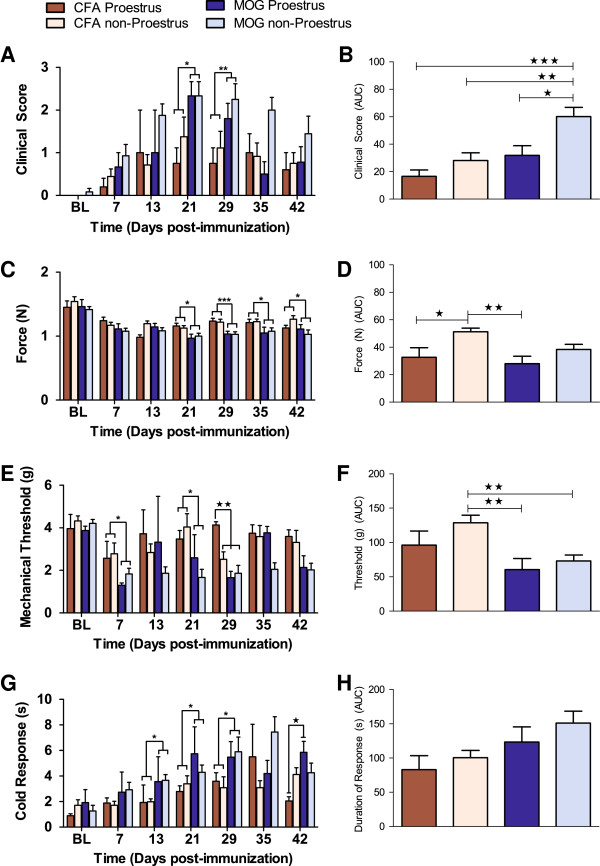
**Proestrus produces protective effects in the EAE model at late time points.** Neurological motor deficits **(A)**, grip strength **(C)**, mechanical withdrawal thresholds **(E)**, and responses to acetone application **(G)** in female mice analyzed across phases of the estrous cycle. Area under the curve (AUC) analyses examining neurological motor deficits **(B)**, grip strength **(D)**, and sensitivity to mechanical **(F)** and cold **(H)** stimulation in females analyzed across the phases of the estrous cycle. Non-proestrus includes the following phases: diestrus, metestrus, and estrus. *BL* baseline. Values represent mean ± SEM. ***P < 0.001, **P < 0.01, *P < 0.05 main effect of treatment as indicated (two-way ANOVA comparing treatment by estrous). ★★★*P* < 0.001, ★★*P* < 0.01, ★*P* < 0.05 comparison as indicated (one-way ANOVA and Bonferroni correction). *N* = 3–16/group, except *n* = 2 in groups CFA proestrus and MOG_35-55_ proestrus on day 13.

Data were analyzed with a two-way RM ANOVA comparing treatment (MOG_35-55_ vs. CFA) and time. For the data of Figure 3, due to the difference in animal numbers (BL–43 days: *n* = 17–18/group; 60–90 days: *n* = 8–9/group, see ‘Methods’ section), separate analyses were conducted for each of these two time intervals (Table 2). If the two-way RM ANOVA revealed a main effect of treatment or treatment-by-time interaction, then one-way ANOVAs were performed to compare treatment conditions at each time point. Estrous data (classified via vaginal lavage, Figure 4) was analyzed with two-way ANOVAs comparing treatment condition (MOG_35-55_ vs. CFA) and estrous phase (proestrus vs. non-proestrus) (Figure 5; Table 3). When the interactions of treatment by estrous were present, a one-way ANOVA was performed followed by a Bonferroni correction. In the small number of instances where estrous phase could not be determined due to technical problems (9 cases out of 245), data could not be considered for the analysis summarized in Figure 5 (this accounts for differences in animal numbers in the statistics shown in Table 3). AUC transformations examining the effects of estrous phase on neurological motor deficits and pain-like behaviors at representative time points, randomly selected at each week of estrous monitoring, were analyzed with a one-way ANOVA.

**Table 2:** Statistics for experiment 2

**Measure**	**Two-way RM ANOVA (treatment × time (BL–day 43))**		**Two-way RM ANOVA (treatment × time (days 60–90))**
Clinical scores	Figure 3A	Day: *F*_37,1221_ = 21.3, *P* < 0.001	Day: *F*_2,30_ = 0.8, *P* = 0.4
		Treatment: *F*_1,33_ = 21.7, *P* < 0.001	Treatment: *F*_1,15_ = 0.003, *P* = 0.9
		Day × treatment: *F*_37,1221_ = 4.1, *P* < 0.001	Day × treatment: *F*_2,30_ = 1.7, *P* = 0.19
Grip strength	Figure 3B	Day: *F*_13,429_ = 17.9, *P* < 0.001	Day: *F*_2,30_ = 4.7, *P* < 0.05
		Treatment: *F*_1,33_ = 22.6, *P* < 0.001	Treatment: *F*_1,15_ = 0.16, *P* = 0.6
		Day × treatment: *F*_13,429_ = 0.9, *P* = 0.5	Day × treatment: *F*_2,30_ = 1.4, *P* = 0.2
Weight	Figure 3C	Day: *F*_36,1188_ = 76.3, *P* < 0.001	Day: *F*_2,30_ = 17.1, *P* < 0.001
		Treatment: *F*_1,33_ = 1.0, *P* = 0.3	Treatment: *F*_1,15_ = 0.5, *P* = 0.4
		Day × treatment: *F*_36,1188_ = 0.5, *P* = 0.9	Day × treatment: *F*_2,30_ = 0.6, *P* = 0.4
Mechanical thresholds	Figure 3D	Day: *F*_13,429_ = 5.5, *P* < 0.001	Day: *F*_2,30_ = 30.4, *P* = 0.06
		Treatment: *F*_1,33_ = 20.9, *P* < 0.001	Treatment: *F*_1,15_ = 0.5, *P* = 0.4
		Day × treatment: *F*_13,429_ = 2.8, *P* < 0.01	Day × treatment: *F*_2,30_ = 1.0, *P* = 0.3
Acetone withdrawal	Figure 3E	Day: *F*_13,429_ = 11.1, *P* < 0.001	Day: *F*_2,30_ = 0.5, *P* = 0.5
		Treatment: *F*_1,33_ = 8.9, *P* < 0.01	Treatment: *F*_1,15_ = 0.2, *P* = 0.6
		Day × treatment: *F*_13,429_ = 2.0, *P* = 0.06	Day × treatment: *F*_2,30_ = 1.6, *P* = 0.2
Heat latency	Figure 3F	Day: *F*_7,231_ = 1.8, *P* = 0.07	**-**
		Treatment: *F*_1,33_ = 1.9, *P* = 0.17	
		Day × treatment: *F*_7,231_ = 0.3, *P* = 0.9	

Treatment conditions: CFA, *n* = 17 (BL–day 43) or *n* = 8 (days 60–90). CFA + MOG_35-55_, *n* = 18 (BL–day 43) or *n* = 9 (days 60–90).

**Table 3:** Statistics for experiment 2 to determine the effects of estrous phase (proestrus vs. non-proestrus phases)

	**Two-way RM ANOVA (treatment × estrous)**						
	**BL**	**Day 7**	**Day 13**	**Day 21**	**Day 29**	**Day 35**	**Day 42**
Clinical scores, Figure 5A							
Treatment	*F*_1,28_ = 0.2, *P* = 0.6	*F*_1, 27_ = 2.0, *P* = 0.16	*F*_1,30_ = 1.09, *P* = 0.3	*F*_1,30_ = 10.3, *P* < 0.05	*F*_1,31_ = 8.5, *P* < 0.01	*F*_1,31_ = 0.5, *P* = 0.4	*F*_1,31_ = 2.2, *P* = 0.1
Estrous	*F*_1,28_ = 0.2, *P* = 0.6	*F*_1, 27_ = 0.5, *P* = 0.4	*F*_1,30_ = 0.2, *P* = 0.5	*F*_1,30_ = 0.6, *P* = 0.4	*F*_1,31_ = 1.1, *P* = 0.2	*F*_1,31_ = 3.0, *P* = 0.09	*F*_1,31_ = 2.8, *P* = 0.053
Treatment × estrous	*F*_1,28_ = 0.2, *P* = 0.6	*F*_1, 27_ = 0.001, *P* = 0.9	*F*_1,30_ = 1.09, *P* = 0.3	*F*_1,30_ = 0.6, *P* = 0.4	*F*_1,31_ = 0.2, *P* = 0.9	*F*_1,31_ = 3.7, *P* = 0.6	*F*_1,31_ = 3.7, *P* < 0.05
Grip strength, Figure 5C							
Treatment	*F*_1,28_ = 0.3, *P* = 0.5	F_1. 27_ = 2.7, *P* = 0.1	*F*_1,30_ = 0.09, *P* = 0.7	*F*_1,30_ = 11.0, *P* < 0.05	*F*_1,31_ = 19.8, *P* < 0.001	*F*_1,31_ = 5.8, *P* < 0.05	*F*_1,31_ = 6.4, *P* < 0.05
Estrous	*F*_1,28_ = 0.04, *P* = 0.8	*F*_1, 27_ = 0.7, *P* = 0.3	*F*_1,30_ = 0.6, *P* = 0.4	*F*_1,30_ = 0.002, *P* = 0.9	*F*_1,31_ = 0.05, *P* = 0.8	*F*_1,31_ = 0.1, *P* = 0.7	*F*_1,31_ = 0.1, *P* = 0.7
Treatment × estrous	*F*_1,28_ = 0.3, *P* = 0.5	*F*_1, 27_ = 0.06, *P* = 0.8	*F*_1,30_ = 2.1, *P* = 0.15	*F*_1,30_ = 0.5, *P* = 0.4	*F*_1,31_ = 0.02, *P* = 0.8	*F*_1,31_ = 0.009, *P* = 0.9	*F*_1,31_ = 0.02, *P* = 0.8
Mechanical thresholds, Figure 5E							
Treatment	*F*_1,28_ = 0.08, *P* = 0.7	*F*_1, 27_ = 4.2, *P* < 0.05	*F*_1,30_ = 0.7, *P =* 0.3	*F*_1,30_ = 7.4, *P* < 0.05	*F*_1,31_ = 25.9, *P* < 0.001	*F*_1,31_ = 2.0, *P* = 0.16	*F*_1,31_ = 3.5, *P* = 0.07
Estrous	*F*_1,28_ = 0.9, *P* = 0.3	*F*_1, 27_ = 0.4, *P* = 0.4	*F*_1,30_ = 2.3, *P* = 0.13	*F*_1,30_ = 0.09, *P* = 0.7	*F*_1,31_ = 5.2, *P* < 0.05	*F*_1,31_ = 3.1, *P* = 0.08	*F*_1,31_ = 0.2, *P* = 0.6
Treatment × estrous	*F*_1,28_ = 0.001, *P* = 0.9	*F*_1, 27_ = 0.09, *P* = 0.7	*F*_1,30_ = 0.1, *P* = 0.7	*F*_1,30_ = 1.5, *P* = 0.2	*F*_1,31_ = 8.7, *P* < 0.01	*F*_1,31_ = 2.1, *P* = 0.14	*F*_1,31_ = 2.6, *P* = 0.11
Acetone withdrawal, Figure 5G							
Treatment	*F*_1,28_ = 0.1, *P* = 0.7	*F*_1,27_ = 1.9, *P* =0.17	*F*_1,30_ = 4.2, *P* < 0.05	*F*_1,30_ = 4.2, *P <* 0.05	*F*_1,31_ = 5.2, *P* < 0.05	*F*_1,31_ = 1.0, *P* = 0.3	*F*_1,31_ = 7.2, *P* < 0.05
Estrous	*F*_1,28_ = 0.01, *P* = 0.9	*F*_1,27_ = 0.000, *P* = 0.9	*F*_1,30_ = 0.01, *P* = 0.9	*F*_1,30_ = 0.02, *P* = 0.6	*F*_1,31_ = 0.001, *P* = 0.9	*F*_1,31_ = 0.8, *P* = 0.7	*F*_1,31_ = 0.09, *P* = 0.7
Treatment × estrous	*F*_1,28_ = 0.9, *P* = 0.3	*F*_1,27_ = 0.06, *P* = 0.7	*F*_1,30_ = 0.001, *P* = 0.9	*F*_1,30_ = 1.2, *P* = 0.2	*F*_1,31_ = 0.2, *P* = 0.6	*F*_1,31_ = 3.7, *P* = 0.06	*F*_1,31_ = 6.3, *P* < 0.05
AUC	Clinical scores:		Grip strength:		Mechanical thresholds:		Acetone withdrawal:
	one-way ANOVA:		one-way ANOVA:		one-way ANOVA:		one-way ANOVA:
	F_3,44_ = 8.8, *P* < 0.001		*F*_3,44_ = 5.3, *P* < 0.01		*F*_3,44_ = 5.8, *P* < 0.01		*F*_3,44_ = 2.8, *P* < 0.05
	(Figure 5B)		(Figure 5D)		(Figure 5F)		(Figure 5H)

Treatment conditions: CFA, *n* = 17; CFA + MOG_35-55_, *n* = 18. Estrous phase: proestrus (*n* = 4–18/day) or non-proestrus phases (*n* = 17–30/day). *AUC* area under the curve.

The Greenhouse-Geisser correction was applied to all RM ANOVAs, where the epsilon value from Mauchly’s test of sphericity was <0.75, and the significance level was *P* < 0.05 (the assumption of sphericity was violated). In these cases where the Greenhouse-Geisser correction factor was applied, degrees of freedom reported reflect the uncorrected values. AUC data were analyzed with one-way ANOVAs followed by Bonferroni correction. SPSS 19.0 (SPSS Inc., Chicago, IL, USA) statistical software was employed. *P <* 0.05 was considered statistically significant.

## Results

### Experiment 1: sex differences in the EAE model

#### MOG_35-55_ produces neurological motor dysfunction in both females and males

##### Clinical scores

As illustrated in Table 1, three-way RM ANOVA comparing sex (female vs. male) by treatment (MOG_35-55_ vs. CFA) by time revealed that MOG_35-55_ produced greater neurological motor impairments (muscle weakness/paralysis) relative to CFA (*P* < 0.001; main effect of treatment) over the 3-week time course (*P* < 0.01; treatment-by-time interaction). Although female EAE mice began to show neurological motor deficits relative to same-sex CFA controls on day 10, whereas male EAE mice did not begin to show these same deficits relative to same-sex CFA controls until day 14, this conclusion is tentative because we found no main effect of sex and no interaction of sex by treatment by time (both *P* > 0.05; Table 1). As illustrated in Figure 1A,B, subsequent two-way RM ANOVAs revealed that bilateral flank injections of CFA produced small neurological motor function deficits in either females or males, such as hanging tail or impaired righting reflex. MOG_35-55_ treatment produced greater impairments relative to CFA controls, including mild paralysis of one or both limbs, in female (*P* < 0.01; Table 1; Figure 1A) and male (*P* < 0.01; Table 1; Figure 1B) mice over the 3-week evaluation period (females, *P* < 0.05; males, *P* < 0.01; treatment-by-time interaction; Table 1).

##### Grip strength

As illustrated in Table 1, three-way RM ANOVA comparing sex (female vs. male) by treatment (MOG_35-55_ vs. CFA) by time revealed that both female and male EAE mice presented with decreased forelimb grip strength when compared to the CFA controls (*P* < 0.01) over the 3-week time course (*P* < 0.05; treatment-by-time interaction). Male mice demonstrated greater forelimb grip strength values relative to females (*P* < 0.001; main effect of sex), consistent with the previous finding that male mice have greater muscular strength than females [38]. However, MOG_35-55_ did not interact with sex: there was neither a sex-by-time interaction, treatment-by-sex interaction, nor a sex-by-treatment-by-time interaction (all *P* > 0.05; Table 1). As illustrated in Figure 1C,D, subsequent two-way RM ANOVAs indicated that MOG_35-55_ treatment, relative to sex-matched CFA controls, did not significantly impact responses in females (*P* = 0.1; Figure 1C), whereas an effect of treatment was evident in males (*P* < 0.05; Table 1; Figure 1D). The trend of MOG_35-55_-induced decreases on grip strength for females at later time points is confirmed in Figure 3A.

##### Body weight

Three-way RM ANOVA comparing sex (female vs. male) by treatment (MOG_35-55_ vs. CFA) by time revealed no differences in weight between MOG_35-55_ and CFA controls (*P* = 0.6; Table 1); as expected, males did gain weight throughout the course of the study at a more rapid rate than their female counterparts (*P* < 0.01; sex-by-time interaction; Table 1). Subsequent two-way RM ANOVAs comparing treatment by time also found no differences in body weight between animals treated with MOG_35-55_ vs. CFA (females: *P* = 0.6, Table 1, Figure 1E; males: *P* = 0.8, Table 1, Figure 1F) over the 3-week time course for either sex.

#### EAE produces mechanical and cold hypersensitivity in female, but not male mice

No study to date has evaluated sex differences in response to mechanical, cold, and thermal stimulation in male and female mice using the MOG_35-55_-induced EAE model.

##### Mechanical hyperalgesia

As illustrated in Table 1, three-way RM ANOVA comparing sex by treatment by time revealed higher mechanical withdrawal thresholds in males than females (*P* < 0.05; main effect of sex) and in MOG_35-55_-treated animals compared to CFA controls (main effect of treatment: *P* < 0.01; interaction of treatment by time: *P* < 0.01). Two-way RM ANOVAs revealed that MOG_35-55_ treatment decreased mechanical withdrawal thresholds in females (*P* < 0.01 vs. CFA controls; Table 1; Figure 2A) from days 11–21 post-immunization (*P* < 0.001; treatment-by-time interaction; Table 1) relative to sex-matched CFA controls; by contrast, MOG_35-55_ did not decrease mechanical withdrawal thresholds in males (*P* = 0.2; Table 1; Figure 2B). One-way ANOVA of AUC for mechanical withdrawal thresholds on testing days 7–21 visually confirms that female MOG_35-55_ mice presented with lower thresholds than either males or sex-matched female CFA controls (*P* < 0.001; Figure 2G).

##### Cold hyperalgesia

Three-way RM ANOVA comparing sex by treatment by time revealed that cold hyperalgesia was significantly impacted by sex, with females demonstrating greater response withdrawal durations to acetone application relative to their male counterparts (*P* < 0.001; Table 1). A treatment-by-sex-by-time interaction suggests that female, but not male, mice receiving MOG_35-55_ demonstrated increased responsivity to acetone following immunization (*P* < 0.001; Table 1). Two-way RM ANOVAs revealed that MOG_35-55_ increased the duration of response following acetone application to the hind paws in female mice relative to sex-matched CFA controls (*P* < 0.05; Table 1; Figure 2C) observed on days 13–21 (*P* < 0.001; treatment-by-time interaction; Table 1); no alterations in response duration were observed in male mice (*P* = 0.8; Table 1; Figure 2D). One-way ANOVA of AUC over testing days 7–21 confirmed that cold response duration was greater in female MOG_35-55_ mice relative to sex-matched CFA controls and males (*P* < 0.001; Table 1; Figure 2H).

##### Heat responses

Three-way RM ANOVA of heat stimulation responses yielded no main effects of either sex (*P* = 0.14; females, Figure 2E; males, Figure 2F; Table 1) or treatment (*P* = 0.8; Table 1). AUC analysis of withdrawal latency to thermal stimulation also failed to yield differences (*P* = 0.4; one-way ANOVA; Table 1; Figure 2I).

### Experiment 2: time course of EAE and effects of estrous state

#### Extended time course of EAE-induced neuropathic pain-like behavior in female mice

The time course of previous studies of neuropathic pain in EAE models are generally limited to 1 month or less, allowing a description of the onset and peak of hyperalgesia, but not remission [24,28]. The one exception is a MOG_35-55_ study that followed mice over a 50-day time course; although mechanical allodynia decreased over this time period, full remission did not occur [23]. To determine the duration of neuropathic pain-like behaviors and their correlation with neurological motor impairment, we repeated our measurements of sensory and motor functions in females—this time with a time course of 90 days—and analyzed the data with two-way RM ANOVA.

##### Clinical scores

As illustrated in Figure 3, MOG_35-55_ treatment induced neurological motor deficits (*P* < 0.001; Table 2; Figure 3A). Motor impairments varied with time (*P* < 0.001; treatment-by-time interaction; Table 2); beginning on day 10 and lasting through day 35, deficits re-appeared for one additional day (day 43). We note a trend of increased neurological motor impairments in mice receiving MOG_35-55_ on days 35–38 (*P* < 0.10 for each comparison). Subsequent evaluations between days 60 and 90 failed to yield differences between the MOG_35-55_ and CFA mice (*P* = 0.19; Table 2).

##### Grip strength

MOG_35-55_ consistently decreased forelimb grip strength (*P* < 0.001; Table 2; Figure 3B) from days 17–42 (*P* < 0.05 for each comparison), with two earlier time points (days 3 and 11) also showing a difference. Later time points failed to yield differences in grip strength between female MOG_35-55_ and CFA mice (*P* = 0.2; Table 2).

##### Body weight

MOG_35-55_ did not alter body weight when compared to CFA controls (*P* = 0.3; Table 2; Figure 3C).

##### Mechanical hyperalgesia

MOG_35-55_ decreased mechanical withdrawal thresholds relative to CFA controls (*P* < 0.001; Table 2; Figure 3D) beginning on day 5 and continuing through day 42 (*P* < 0.01; treatment-by-time interaction; Table 2), with no differences over days 60–90 (*P* = 0.3; Table 2).

##### Cold hyperalgesia

MOG_35-55_ increased the responsiveness to topical acetone application (*P* < 0.01; Table 2; Figure 3E) beginning on day 7 and lasting through day 42 (*P* < 0.05 for each comparison), with no differences over days 60–90 (*P* = 0.2; Table 2).

##### Heat responses

Compared to the CFA controls, MOG_35-55_ did not produce heat hypersensitivity (Table 2; Figure 3F) through day 43 (*P* = 0.9); therefore, heat testing was discontinued.

#### EAE is associated with fewer neurological motor deficits during proestrus

Little is known about the relationship between estrous cycle and neuropathic pain-like behaviors in animal models of MS. To address this question, we evaluated mechanical and cold allodynia while monitoring the estrous cycle. As illustrated in Figure 4, female mice that received either CFA (Figure 4A) or CFA + MOG_35-55_ (Figure 4B) demonstrated prolonged periods of time spent in the diestrus phase as classified by vaginal lavage. This is consistent with previous reports indicating that intraplantar CFA prolonged the leukocytic phase of the estrous cycle [39]. Within approximately 14 days after initial CFA or CFA + MOG_35-55_ administration, estrous cycling returned to normal. The CFA-induced prolongation of diestrus resulted in unequal representation of the estrous phases within our study. Therefore, in order to investigate effects of the phase with the greatest hormonal fluctuations, we chose to bin our analyses into proestrus (progesterone, estradiol, and luteinizing hormone surge) and ‘non-proestrus’ phases (diestrus, metestrus, and estrus). The effect of estrous phase on neurological motor deficits and pain-like behaviors were further analyzed using two-way ANOVA. Though the treatment condition (MOG_35-55_ vs. CFA) was consistent at each time point, the animals classified as being within proestrus or non-proestrus was not constant. Thus, the number of animals in each of the different phases of estrous varied at each post-immunization time point. Therefore, it was not possible to analyze our data by RM ANOVA with time as the repeated measure.

##### Clinical scores

As illustrated in Figure 5A,B, two-way ANOVAs at each time point confirmed that MOG_35-55_, compared to CFA controls, produced neurological motor deficits, significantly on days 21 and 29 (*P* < 0.05; Table 3). We found an estrous-by-treatment interaction on day 42 (*P* < 0.05; Table 3), but when the same data were subjected to a one-way ANOVA, no significance was noted (*F*_3,34_ = 1.0, *P* = 0.3). One-way ANOVA of all time points, transformed as AUC, suggests that proestrus was protective against neurological motor deficits in MOG_35-55_ animals (*P* < 0.001; bottom row of Table 3); however, this conclusion is presented with caution as we did not find a significant main effect of estrous at any particular time point (*P* > 0.05).

##### Grip strength

MOG_35-55_ reduced forelimb grip strength at several time points (day 21: *P* < 0.05; day 29: *P* < 0.001; day 35: *P* < 0.05; day 42: *P* < 0.05; Table 3). However, grip strength did not change with estrous cycle at any particular day (effect of estrous: *P* > 0.3 for all time points, Table 3; estrous-by-treatment interaction *P >* 0.15 for all time points, Table 3) (Figure 5C). Analysis of grip strength data using AUC revealed that proestrus was associated with lower grip strength values as compared to non-proestrus in CFA controls (*P* < 0.01; one-way ANOVA; Figure 5D).

##### Mechanical hyperalgesia

MOG_35-55_ reduced mechanical thresholds on days 7, 21, and 29 (*P* < 0.05; Table 3). When the data was transformed as AUC, we found a main effect of MOG_35-55_ (*P* < 0.01; Table 3; Figure 5E,F)_._ On day 29, two-way ANOVA revealed an effect of estrous (*P* < 0.05; Table 3) and an estrous-by-treatment interaction (*P* < 0.01; Table 3; Figure 5E); however, we caution against over-interpretation of this result since this occurred at just one time point, and AUC transformation yielded no effect of estrous over multiple testing days.

##### Cold hyperalgesia

MOG_35-55_ increased the response to cold stimulation on days 13, 21, 29, and 42 (*P* < 0.05; Table 3; Figure 5G). Although we found an interaction of treatment by estrous on day 42 (*P* < 0.05; Table 3), the conclusions are tentative as there was no main effect of estrous on cold stimulation at any particular time point (*P* > 0.6 for each time point; Table 3). A subsequent one-way ANOVA revealed that cold hyperalgesia was greater in the MOG_35-55_-proestrus animals as compared to CFA-proestrus animals on day 42 (*F*_3,34_ = 3.7, *P* < 0.05). When all time points were transformed as AUC, we found a main effect of group (*P* < 0.05; Table 3; Figure 5H); however, subsequent *post hoc* analysis did not reveal significant differences between groups (*P >* 0.05 for each comparison).

## Discussion

To date, no study has rigorously characterized the effects of sex and estrous state on the development, duration, and severity of pain symptoms associated with MOG_35-55_-induced EAE. Our present study addressed this gap with behavioral assessment of sensory and motor functions in C57BL/6 female mice for 3 months after induction of EAE with MOG_35-55_ immunization and compared select time points with males. We report four general findings. First, in experiment 1, MOG_35-55_ produced neurological motor impairments in both females and males, including mild paralysis of hind limbs and decreases in forelimb grip strength (particularly in experiment 2). Second, MOG_35-55_ produced mechanical and cold hypersensitivity only in females. Third, the duration of pain-like behavior in 3–4-month-old (at study onset) C57BL/6 female mice in experiment 2 was 42 days. Subsequent studies using younger female mice have yielded much longer durations of pain-like behavior (unpublished observations from our laboratory). Fourth, experiment 2 revealed that MOG_35-55_ produced less neurological motor dysfunction when female mice were in the proestrus phase.

### Sex differences in the clinical manifestations of EAE

Multiple sclerosis is a disease dominated by female patients with a 2:1 prevalence in females relative to males. Our study tested the hypothesis that neurological motor impairment would be greater in females in the most commonly utilized animal model of MS, the mouse MOG_35-55_ EAE model [21,28,40]. As described previously, we found that MOG_35-55_ produced motor dysfunction, characterized by mild paralysis of one or both hind limbs [28], for review see [41]. And, in agreement with one previous study [33], we found that MOG_35-55_ decreased grip strength, an effect most evident 2–3 weeks after initial immunization. This robust behavior persisted in females for up to 42 days post-immunization (Figure 3B).

Similar to the results of Okuda and colleagues, we found that MOG_35-55_ produced motor impairments and decreases in grip strength in both male and female C57BL/6 mice, indicating an absence of sex differences in EAE severity; however, in our study, females developed neurological deficits as reported with a clinical scoring assessment 4 days prior to the development of such deficits in males when the mice are compared to same-sex CFA controls, whereas they reported no difference in onset of clinical deficits [20]. By contrast, female SJL and ASW mice develop more severe clinical symptoms than males when treated with the EAE-inducing encephalitogenic peptides myelin basic protein (MBP) and proteolipid protein (PLP), respectively [21]. Conversely, males develop more severe neurological deficits (bilateral hind limb paralysis) in the TMEV model, a mouse model of progressive MS [22]. Further studies are needed to determine the importance of immunization protocol, dose of adjuvant, mouse strain, age, and other factors on sex differences in murine models of MS.

### Sex differences in EAE-induced mechanical and cold hypersensitivity

MS is one of many disease states which show a particularly high prevalence of pain in women [42], necessitating investigation of sex-associated hypersensitivity in preclinical MS models. Our study is the first to compare EAE-associated nociception between male and female mice using a MOG_35-55_ immunization protocol. For two reasons, we feel it unlikely that these pain-like behaviors were indirectly inhibited by concomitant neurological motor deficits. First, we found that pain-like behaviors occurred prior to the onset of clinical signs, in accordance with previous observations. Second, we observed robust nociceptive responses despite motor deficits.

As observed previously by Olechowski and colleagues [28], we found that MOG_35-55_, as compared to sex- and age-matched CFA controls, produced hypersensitivity to mechanical and cold stimulations in 3–4-month-old C57BL/6 female mice. In females, hyperalgesia was present up to 42 days following initial immunization, and subsided by 60 days, with no relapse noted by day 90. Remarkably, we did not observe hyperalgesia in their male MOG_35-55_ counterparts. Our results are consistent with the finding that female SLJ/J mice in the TMEV model of multiple sclerosis exhibited a faster onset and greater peak of mechanical allodynia as compared to males [22,43]. Similarly, heat hyperalgesia was more pronounced in females in the PLP model of EAE in SJL mice [24]. Our study did not reveal heat hypersensitivity, perhaps due to different localization of the heat stimulus in our study (hindpaw) vs. the previous study (tail and forepaws) [24]. Olechowski and colleagues also reported a lack of thermal hyperalgesia in MOG_35-55_ females that nevertheless demonstrate robust mechanical and cold allodynia [28].

### Duration of pain-like behaviors in MOG_35-55_-induced EAE

To date, no study has examined neuropathic pain-like behaviors beyond 50 days, when minor mechanical allodynia was noted in female subjects [23,24,28]. Here, we report that mechanical and cold hyperalgesia may antedate, but do not outlast MOG_35-55_-induced neurological motor impairments. Both neuropathic pain-like behaviors and neurological motor impairments were present up to 42 days post-immunization, and we failed to observe a relapse; however, further assessment beyond 90 days is needed to determine whether our model reflects the relapsing-remitting form of EAE.

### Neurological motor impairment is dampened during proestrus

The lack of information regarding the relationship between neurological deficits, pain behaviors, and estrous cycle prompted our investigation of cycle-related alterations in the sensory and motor disturbances associated with EAE. In experiment 2, we observed that CFA administration alone, or in combination with MOG_35-55_, prolonged the time spent in diestrus, thereby halting normal estrous cycling for 2 weeks—consistent with a previous report [39]. Strikingly, neurological motor deficits measured via daily clinical assessments were attenuated during proestrus as compared to the other phases; this protection was only evident with AUC transformations that allowed us to examine the more subtle effects of estrous phase over multiple testing days. Because proestrus is characterized by relatively high plasma levels of estrogen and progesterone [44], our results are consistent with the hypothesis that circulating ovarian hormones decrease neurological motor deficits in female MOG_35-55_ mice. Progesterone administered prior to EAE immunization delayed onset and attenuates progression of neurological deficits [45]. Work in the EAE models has also demonstrated the possible role of estrogens/progesterone in promoting remyelination and reducing the presence of pro-inflammatory mediators (e.g., TNF-α) and microglial activation [46-50], providing potential mechanisms through which ovarian hormones may produce protection against neurological motor deficits.

We were unable to conclusively demonstrate estrous-related alterations in pain behaviors. A veritable host of studies have investigated sex- and hormone-related alterations of somatosensation in other preclinical models of chronic pain, with conflicting findings. Some studies report greater hyperalgesic responses in females during the proestrus phase [51,52], while others report antinociception associated with the proestrus phase [53] or with the exogenous administration of estrogens [54-56]. More investigations are necessary to determine how natural and directed alterations in hormone levels may impact EAE pain-like behaviors.

While our study is the first to report on estrous cycle alterations in EAE-induced neurological motor dysfunction, our findings do have important commonalities with clinical MS populations. A recent report examined menstrual cycle effects on MS symptoms and found that women taking oral contraceptives experienced increased weakness, numbness, and tiredness during their menstrual cycle when not taking oral steroids [3]. A similar study reported menstruation-related increases in MS symptoms, including pain, in normal cycling women [5]. The onset of menses is associated with low levels of estrogen and progesterone [57]; therefore, these studies collectively suggest a worsening of symptoms when ovarian hormones are low and, conversely, a potentially protective effect when levels are high. These clinical studies parallel our findings in the EAE model and point to the clinical therapeutic potential offered from further investigations into mechanisms mediating the protective and antinociceptive effects of ovarian hormones in the EAE model.

It is well established that clinical symptoms of MS are attenuated during pregnancy, likely due to elevations in estrogens and progesterone. This is followed by an increased risk of postpartum relapse when levels of estrogens and progesterone are low [6,58]. Similarly, studies in pregnant SJL and C57BL/6 mice reported pregnancy-associated improvement in EAE symptoms and a subsequent increase in postpartum relapse, concomitant with decreased estrogen levels [59,60]. Further, administration of estriol, an estrogen produced only during pregnancy, ameliorated symptoms and lesions as assessed by magnetic resonance imaging in non-pregnant female MS patients [61] and clinical impairments in EAE mice [62,63]. It remains to be investigated if pregnancy-induced remission in EAE symptoms would include reduction of hypersensitivity to cutaneous and cold stimulation, in addition to the possible analgesic role that exogenously applied estrogens/progesterone may play.

## Conclusions

In agreement with previous reports [24,28], for review see [64], we conclude that the MOG_35-55_ EAE model induces neurological motor dysfunction and neuropathic pain-like behavior, similar to symptom profiles observed in clinical MS populations. While male C57BL/6 mice develop neurological motor impairments typical of the EAE model, they fail to develop the neuropathic pain-like behaviors observed in their female counterparts. EAE-induced neurological motor impairments were reduced during the proestrus phase, a finding that warrants further investigations into the contributing role of circulating ovarian hormones to EAE pathology. We suggest that female C57BL/6 mice receive preference in future studies of neuropathic pain-like behaviors associated with the MOG_35-55_ model of EAE. Care must be taken to consider the effects that estrous cycling may have on neurological motor deficits.

## Abbreviations

AUC: area under the curve; CFA: complete Freund’s adjuvant; EAE: experimental autoimmune encephalomyelitis; MOG35-55: myelin oligodendrocyte glycoprotein 35–55; MS: multiple sclerosis; PLP: proteolipid protein; TMEV: Theiler’s murine encephalomyelitis virus.

## Competing interests

The authors declare that they have no competing interests.

## Authors’ contributions

EJR helped conceive the study; carried out vaginal lavages, all behavioral evaluations, and immunizations; and helped draft the manuscript. TI helped draft the manuscript. RRD assisted in vaginal lavage and immunizations. BKT helped conceive the study, participated in its design and coordination, and helped draft the manuscript. All authors read and approved the final manuscript.

## Authors’ information

For the research description and interests of BKT, please refer to https://physiology.med.uky.edu/users/bkta222.
